# The future demand of renewable fuels in Germany: Understanding the impact of electrification levels and socio-economic developments

**DOI:** 10.1016/j.heliyon.2023.e22271

**Published:** 2023-11-13

**Authors:** Jonathan Vincents Eriksen, Sebastian Marco Franz, Julius Steensberg, Adam Vejstrup, Mikkel Bosack, Rasmus Bramstoft, Fabian Scheller

**Affiliations:** a Department of Technology, Management and Economics, Technical University of Denmark (DTU), Denmark; b Energy Modelling Lab (EML), Denmark; c Institute Zero Carbon (IZEC), Technical University of Applied Sciences Würzburg-Schweinfurt (THWS), Germany; d Center for Applied Energy Research (CAE), Würzburg, Germany

**Keywords:** Renewable fuels, Hydrogen, Energy system analysis, Energy transition, Shared socioeconomic pathways

## Abstract

The Climate Change Act recently enacted in Germany highlights the urgency of understanding the future demand for renewable fuels. In this study, we combine technological progress and socio-economic pathways in an energy system analysis to assess future renewable fuel demands in Germany. We apply the whole-system optimisation model, TIMES, to investigate transition pathways with varying electrification levels and socio-economic developments. The results show that renewable fuels demand varies between 388 PJ and 1310 PJ depending on the electrification rates. Furthermore, our findings demonstrate that considering socio-economic aspects and behavioural change, as represented by different Shared-Socio-economic Pathways, can significantly alter the demand for renewable fuels within a narrower yet still noteworthy range compared to the electrification scenarios. This provides country-level evidence highlighting the often-overlooked influence of social developments on demand projections. Consequently, it becomes crucial to prioritize the consideration of the climate mitigation potential arising from socioeconomic-induced changes in demand patterns within the broader framework of energy efficiency measures.

## Highlights

• Integration of technological advances with socio-economic pathways in energy analyses.
• Application of TIMES model to forecast Germany's renewable fuel needs.
• Electrification and socio-economic pathways notably affect renewable fuel demand.
• Emphasize the role of socio-economic shifts in shaping demand for robust climate mitigation.

## Nomenclature

**EV**: Electric Vehichle
**GDP**: Gross Domestic Product
**GHG**: Greenhouse Gas
**GLF**: Green Liquid Fuels
**IIASA**: International Institute for Applied System Analysis
**IPCC**: Intergovernmental Panel on Climate Change
**Pkm**: Person kilometre
**RE**: Renewable Energy
**RF**: Renewable Fuel (including E-fuels, biofuels and hydrogen)
**SSP**: Shared Socioeconomic Pathways
**TIMES**: The Integrated MARKAL-EFOM System
**TIMES-DE**: The Integrated MARKAL-EFOM System model for Germany
**TFEC**: Total Final Energy Consumption
**TPES**: Total Primary Energy Supply

## 1 Introduction

In 2021, Germany set a revised, ambitious goal to become climate neutral by 2045 [1], and thereby reinforced the significance of the fulfilment of the Paris Agreement [2]. In addition to the National Hydrogen Strategy of 2020, [3], it stands clear that the future of renewable fuels (RF) as defined by [4], being electro-fuels (E-fuels) and biofuels (aggregated into green liquid fuels, GLF), and green hydrogen in the German system is quite significant. Despite the fact that the current consumption of 200 PJ of hydrogen [3] mainly consists of fossil-derived grey hydrogen, the Federal Government expects a consumption of 325-400 PJ of RFs in 2030 at a point in time where the reduction in carbon emissions are of importance. These ambitious targets are endorsed by studies concluding that the share of RF in the total final energy consumption will rise from 0.2% in 2020 to 10% in 2050 worldwide [5], and to above 20% in European countries [6]. The positive relationship with the decarbonisation targets is also given since RFs have a higher share in Germany's final energy consumption in scenarios with stricter climate targets [7].

The potential of RFs is very broad, and they may prove to replace fossil fuels in the sectors currently consuming gas. This is in the form of pure hydrogen gas or renewable gasses in high-temperature heating in the industry sector [8], while their role in electricity and district heating purposes are less significant [9]. Furthermore, RFs are key components in the decarbonisation of the heavy transport sector [10], [11] such as aviation [12] and shipping [13], as they currently show small potentials of electrification [14]. When dealing with production of renewable transport fuels, it is important to assess an extensive catalogue of RF production pathways [15], including E-fuels [16]. While energy transition of the German power system has been analysed [17], [18], using different energy system modelling tools [19], we focus in this paper on the industrial and transportation sectors, as the main future consumers of RFs [20].

The demand of RFs in the future is highly dependent on various socio-economic aspects [21] and the progression made on the electrification of vehicles and process heat in the industry as, e.g., presented by Dena & ewi (2018) [22]. The Dena et al. [22] study includes two relevant scenarios, which either assume a rapid and extensive electrification of the energy system or assume that the final energy consumption can be met by using a variety of technologies. The extensive electrification scenario is called Electrification 95 (EL95) and assumes a 95% reduction of CO_2_ emissions relative to 1990. The technology mix scenario is named Technology Mix 95 (TM95) and has the same emissions targets as the EL95 scenario. The TM scenario assumes a large development in efficiency and price for a range of technologies, which leads to the significant usage of RFs in the final energy mix relative to the EL95 scenario. TM95 finds a demand for synthetic energy carriers (defined as hydrogen and GLFs) of 3269 PJ, whereas the EL95 scenario computes a demand of 1919 PJ by 2050, of which 2120 PJ and 1166 PJ, respectively are demanded by the transport and industry sector.

However, a lot of progress is currently being made on the electrification of transport [23] [24]. In this context, Prognos & Öko-Institut & Wuppertal-Institut (2020) [25] assume a greater share of electrification in the transport sector than any of the previously discussed scenarios by Dena et al. The KN2050 scenario models a completely carbon-neutral energy system by 2050 and finds a demand for hydrogen and GLFs of only 1555 PJ by 2050, of which 968 PJ is demanded by the industry and transport sector [25].

Table 1 summarises the most relevant findings of these studies in comparison with respect to the current paper, where RF includes hydrogen and GLFs. While both the Dena et al. [22] and Prognos et al. [25] studies use investment models to find the cheapest way to fulfil a final demand, other models like the TIMES model [26] fulfils end-use service demands. This means that the energy system model is free to choose how to cover the end service demand, which could be through technologies using hydrogen or other fuels.

**Table 1:** RFs demand found within studies of comparison. RF includes hydrogen and GLFs. EL 80, EL 95, TM 80 and TM 95 are from [22] and KN 2050 from [25].

Investigation Goal	Year	EL95	TM95	KN2050
Final energy consumption [PJ]	2030	7013	7621	7500
	2050	5306	5749	5700

Consumption of RFs [PJ]	2030	169	166	230
	2050	1919	3269	1555

Consumption of RFs in industry and transport [PJ]	2030	169	166	230
	2050	1166	2120	968

Share of RFs imported [%]	2030	6	2	70
	2050	74	82	81

Share of RFs in final energy consumption [%]	2030	2	2	3
	2050	36	55	27

Share of non-blended H2 to total RF consumption [%]	2030	100	100	98
	2050	32	19	62

The total energy demand and, thereby implicitly the RF demand may follow different pathways, where a scenario of decreased demand seems interesting. Political measures and changes in socioeconomic dynamics could lead to a decrease in demand. For example, the energy saving plan of 2022 [27] and higher energy prices could lead to lower demands [28]. Additionally, a decrease of the demand could also be caused by changes in socioeconomic dynamics, which for example could be a shift of diet towards a higher extent of plant-based, leading to an ease of demand within agriculture [29] or to more comprehensive utilisation of public transport, lowering the total demand of transport [30]. These factors are yet to be applied to Germany, but Gaur et al. have among others investigated the impact of a behaviour change with the TIMES modelling of Ireland [31]. Various experts have also suggested the inclusion of behavioural aspects in energy system analysis as a crucial development [32].

The objective of this study is to examine the future demand for RFs, particularly hydrogen, in Germany for the years 2030 and 2050. To achieve a more comprehensive understanding of the impact of electrification levels and societal dynamics on RFs demand, we applied the energy optimisation model TIMES focusing on the industry and transport sector in Germany. We aim to show that energy efficiency measures induced by socioeconomic pathways have an impact on energy savings similar to the technological progress, such as electrification. This counters the belief that climate mitigation should solely rely on technological progress and highlights the need for a more comprehensive approach that includes behavioural changes.

While the TIMES model has been widely used in analysing the future demand for RFs in various countries such as Norway [33], Italy [34], Denmark [26], and as a larger model investigating 28 EU countries [35], we developed a TIMES-DE version. This allowed us to focus on the specific case of Germany with the appropriate level of detail. Such studies are rather abundant. In contrast to studies by Dena et al. [22] and Prognos et al. [25] which have already focused solely on the coming RF demand of Germany, TIMES provides end-service demand-driven parameters. As Germany is currently a net importer of energy, the promotion and adoption of RFs is relevant not just for Germany but also for neighbouring countries, particularly in the Nordic region.

In addition, existing studies estimating the RF demand of Germany do only include different electrification levels of different sectors but do not investigate the effects of socioeconomic dynamics such as [36]. We utilised the Shared Socioeconomic Pathways (SSP) projections of the IPCC [37] to model future energy demand dynamics based on SSP-specific GDP and population forecasts. Our results, which examine both the production and consumption of RFs, as well as the split of different RFs, can be compared with other studies in this field, adding to the understanding of the future demand for RFs in Germany. Thereby, our contribution to the ongoing discourse on the impact of different socioeconomic pathways on RF demand is significant. This allows us to understand climate mitigation challenges for especially hard-to-abate sectors (e.g. heavy industry, aviation or shipping) and the economy in general and offers a unique perspective on German RF demand dynamics.

## 2 Research methodology

### 2.1 General structure of the TIMES model

TIMES (The Integrated MARKAL-EFOM System) is an energy system optimisation model that utilises a bottom-up approach. It is used for determining the least-cost solution by meeting all energy demands while simultaneously respecting given system constraints. TIMES has a variety of applications and can be applied at various levels of spatial, temporal, and sectoral resolutions [38]. The objective of the model is to minimise total system costs, which is defined as the sum of discounted investments, fixed and variable costs, fuel import costs, and energy export revenues, less the salvage values of investments for which the whole lifetime exceeds the model's time horizon. Further information on TIMES can be found in the TIMES Documentation [39] and the TIMES-DK paper [26].

In TIMES, least-cost optimisation is based on exogenously given inputs and a user-defined scope. The energy system is represented by a flow architecture, where the energy flows (carriers or rather commodities) between processes (system technologies or loads), which consume and produce energy, and emissions such as CO_2_ are defined [40]. The optimisation in TIMES is driven by end service demands, such as mobility in the dimension of passenger kilometres in private cars, heat in the units of PJ input in industry, or a specific amount of tonnes km for freight trains. These end service demands are over time affected by macroeconomic drivers such as GDP and population [41].

On the supply side, an extensive catalogue of technologies are encompassed using techno-economic parameters and future projections of them. Mention-worthy is future availability and techno-economic parameters of ammonia ships or short-distance electric airplanes or a certain degree of electrification of process heat in the industry. Furthermore, respective energy technologies are subject to availability factors in specific types of hours of the year, further defining the feasibility of different technologies. Finally, scenario constraints are implemented to shape a defined scenario, such as a maximum limit on greenhouse gas (GHG) emissions in order to analyse pathways to net-zero carbon societies.

**Figure 1:**
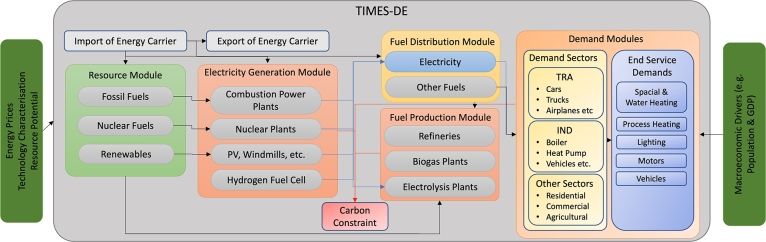
General structure of TIMES-DE. TRA = transport sector, IND = industrial sector, RES = residential sector, COM = commercial sector, AGR = agricultural sector. Layout inspired by [31] and [42].

Fig. 1 presents the general, simplified structure of our TIMES-DE modelling framework as applied in this study. The supply side consists of domestic resources, conversion technologies but also the possibility of importing or exporting various energy carriers. The demand side includes sector-specific processes that use compatible carriers or fuels to satisfy certain demanded services, such as space heating for houses, distances covered in private cars or process heat in the steel industry. While the applied TIMES structure is similar to other models in the literature like [42] or [26], we parameterised a TIMES-DE model in order to represent the German energy system. Thereby, the layout of our figure is inspired by [31] and [42].

### 2.2 Modelling of the transport and industrial sectors in TIMES-DE

**Figure 2:**
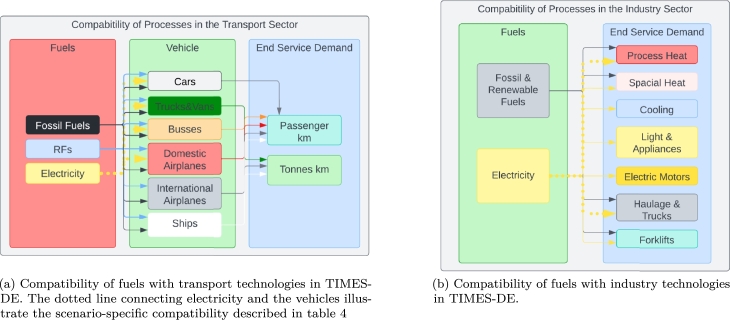
Visualisation of fuels and energy carriers that can supply demands in the transport and industrial sectors. The figures furthermore details which fuels that are in competition with each other for satisfying specific end service demands.

The transport and industrial sectors are known to be hard to fully electrify [8] [10], making them of specific interest when assessing the future demand of RFs, and in particular hydrogen. To provide more detail on these sectors, Fig. 2 presents an overview of the architecture for modelling in TIMES-DE.

Fig. 2a illustrates the various fuels and energy carriers that are in competition to supply the end service demands of the transport sector. Different transport modes have varying fuel requirements and the model optimises the least-cost supply strategy, taking into account the competition and the constraints of the end-service demand.

Similarly, Fig. 2b outlines the competition of supply options to satisfy the end-service demand of the industrial sector. This encompasses a variety of end-use demands, from high-temperature process heat, to low-temperature heat demand and even cooling demand. Furthermore, the electricity consumption is covered as well as the need for transportation inside industries, e.g., using forklifts or trucks. The model optimises the least-cost supply strategy considering the competition between the supply options. Within the optimisation, the possibility of a certain degree of electrification of trucks, vans and domestic air traffic as well as process heat in the industry is included. This is further specified in section 3.5.2.

## 3 Main data and scenario assumptions

### 3.1 Spatial and temporal scope

**Figure 3:**
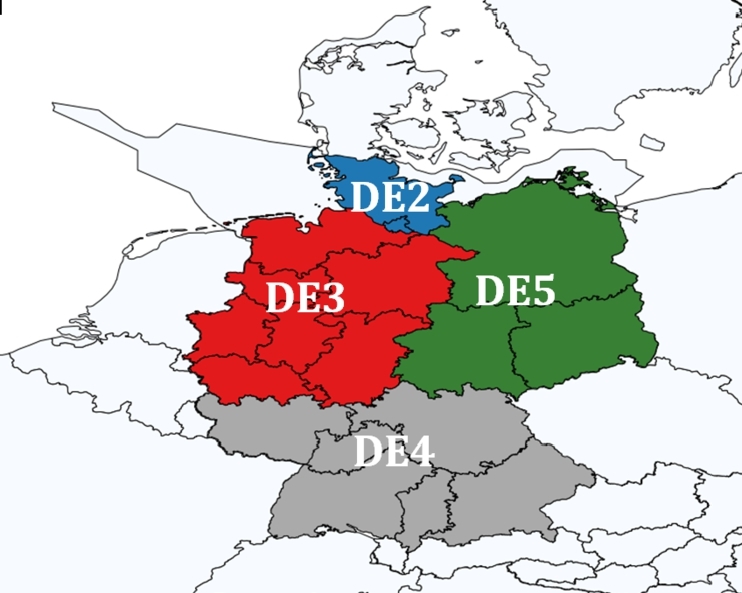
Regional splitting of Germany due to bottlenecks of the electricity transmission. The division of Germany is based on the data from the Balmorel model [44].

This study focuses on the future German demand for RFs. The geographical scope of this study is therefore Germany, with a spatial division to capture internal bottlenecks in the electricity transmission system and to represent the large RE production in the Northern part and the high consumption in South-West region [43]. The specific regional division is adopted from the large-scale energy system model, Balmorel [44], and is illustrated in Fig. 3. However, the consumption of the different sectors is aggregated into a national level following the approach by Contaldi et al. [34]. The end service demands therefore, only exist at the national level, while electricity generation is defined to be in one of the four regions. Flow ratios for the electricity from the regions to the national region have been set to mimic the transmission bottlenecks. Transmission between the regions is possible, but with loss and transmission costs.

The temporal resolution applied in this study is associated with the long-term modelling horizon and the aggregation of time within a simulation year. For the long-term planning horizon, we optimise the least-cost pathway for the German energy system until 2050 in 5-year time intervals. For the time aggregation within a simulation year, the TIMES-DE model aggregates the hourly data into 56 time steps based on the 4 seasons, 2 day types and 7 hour types, as outlined in Table 2. Similar structure has been observed in the TIMES-DK study [26].

**Table 2:** Time slice type definitions. High equals the top 15% and low is the lower 15%. In total there are 4 ⋅ 2 ⋅ 7 = 56 different time slices. The time slice structure is based on the TIMES-DK study [26], with three more hour types in TIMES-DE.

Season types	Day types	Hour types	Hour type ranking
Summer	Working day	Day	1
Fall	Not-working day	Night	2
Winter		High demand	3
Spring		Low wind	4
		High wind and low demand	5
		Low wind and high demand	6
		High solar	7

### 3.2 Existing energy system and techno-economic parameters

The majority of the current energy system data is implemented based on energy balances recorded by Eurostat [45]. In addition, more detailed data is used when available, e.g. electricity and district heat generation capacities. The electricity and district heat generation capacities are adopted from Balmorel model [44], [46], which has preprocessed data of around 900 conventional and one million renewable power plants [47].

Energy balances regarding international shipping of goods were not present in the database of Eurostat. Therefore, the quantities regarding shipping have been calculated from data given at the website of the Port of Hamburg [48], stating the top 10 destinations for vessels from Hamburg, ranked by the loading weight of the shipped containers. This number could then be multiplied by the distance to these harbours. Moreover, data on number of person kilometre (Pkm) travelled in aviation were not available. Therefore, the demand of aviation in the base year has been calibrated to match the consumption of kerosene, taking into account the efficiency of the technology of aviation. TIMES-DE does not encompass the fertiliser demand for the agricultural sector since the model is strictly limited to only take energy commodities into account.

The techno-economic data regarding energy technologies are extracted from the Danish Energy Agency [49] who continuously release technology catalogues containing technical, economic and environmental specifications of energy technologies already in use or at stages of different levels of development. In addition hereto, the future costs and efficiencies of energy technologies are projected in the energy technology catalogues and are used as input in the TIMES-DE model as well.

### 3.3 Resource potentials and profiles

Potentials for renewable energy (RE) sources are incorporated into the TIMES-DE model. The potentials of onshore wind in Germany is 160 GW, and solar PV 380 GW, which is based on data from [50]. The potential was given for the whole of Germany, so each of the four regions in TIMES-DE, see Fig. 3, is assigned a quarter of the total potential. The total offshore wind potential in TIMES-DE is set to 60 GW also based on [50].

In light of the EU Green Deal concerning biomass [51] as well as the indirect effects of land use change as proposed by the Danish Energy Agency [52], it is considered that the future import of biomass will be very restricted, and therefore the import of biomass and biofuels is not possible within TIMES-DE. The German biomass potential is extracted from the S2Biom project [53].

Regarding hydro plants capacity, it is estimated that Germany can not make future expansions, since it is seen from historic input data that hydro power production has decreased slightly since 2011 [54] [55].

Profiles for variable RE generation are based on regional hourly weather data from 2019 gathered from RenewablesNinja [56]. This was done by finding a representative location in each of the four regions and collecting data from these specific points. The availability profiles for wind and solar resources are assumed to be identical for all simulated years, even though there is an uncertainty when it comes to how the weather will change over the next decades [57].

The electricity consumption data was collected from European Network of Transmission System Operators (ENTSO-e) [58]. It was not possible to get hourly consumption data divided on each of the sectors, which results in similar hourly electricity consumption profiles for the five demand sectors.

All the hourly profiles for RE generation and consumption are adopted for the year 2019. This year was chosen based on its characteristics as being a representative year regarding consumption and availability of RE generation. The hourly profiles are aggregated into time slices according to the aforementioned aggregation approach.

### 3.4 Discount rate, fuel prices and emission constraints

A discount rate of 4% is applied, which is based on recommendations for energy system analysis set forth by Garcia-Gusano et al. [59].

Import prices of fuels are based on Analyseforudsætninger 21 [60], whereas the marginal price of fuels within the model is calculated based on technology specifications, input prices and scale.

Finally, the decarbonisation path towards a lower emission society is driven by a limit of GHG emissions. This GHG emission limit is assumed to be linearly interpolated between the 1990 level and a 65% reduction in 2030, and towards net zero emission in 2045, as put forth in the Climate Change Act [1].

### 3.5 Scenarios

#### 3.5.1 Scenario characteristics: energy efficiency and future demands

We use the Shared-Socioeconomic-Pathways (SSP) framework, which is being implemented via different SSP-specific GDP and population forecasts, to model future dynamics in demands. The different SSP-specific futures are used not only to include the necessary socioeconomic and behavioural components but also to use them as a proxy for energy efficiency in a broader sense. This energy efficiency covers both technological advancements but also societal and behavioural measures to improve energy efficiency. This can be seen already in today's society given the tremendous impacts of geopolitical circumstances on the German energy system and, thus, rising energy bills that already lead to significant energy efficiency improvements mainly induced by the behaviour of the respective consumers [61], [62]. We use projections from the International Institute for Applied System Analysis (IIASA) database [63]. The SSP database by IIASA is widely used in energy modelling and in the IPCC Sixth Assessment Report [64]. The various energy demands then follow the relative development of either the German GDP or population. The drivers are similar across all three SSP scenarios, and can be seen in Table 3. The drivers are based on the choices made in the TIMES Ireland Model (TIM) by Gaur et al. [31].

**Table 3:** Energy service demand in different sectors for the base year and estimated for 2050. The main driver for the future demand is indicated for each of the energy service demands and is either GDP or population inspired by the method from [31]. The values presented in the table is associated to SSP2.

Energy service demand	Driver	Values for SSP2		Unit
		2010	2050	
Passenger travels	GDP	1108048	1873031	MPkm
Freight	GDP	6677179	9979049	Mtkm
Industry	GDP	2333	2507	PJ
Agriculture	Population	54	77	PJ
Residential heating	Population	3558	5998	Mm2
Residential cooling	GDP	3558	5998	Mm2
Residential other energy demand	Population	740	663	PJ
Commercial heating and cooling	GDP	411	575	Mm2
Commercial other energy demand	GDP	547	737	PJ

**Figure 4:**
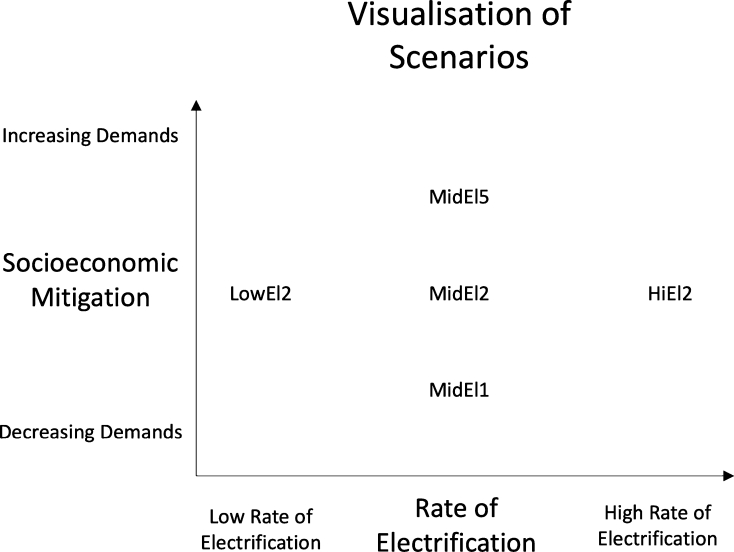
Illustration of the electrification rate and demand projections as implemented in each scenario.

For the projections, we utilise the SSP narratives SSP1, SSP2 and SSP5. The reason for having several projections is to more thoroughly investigate the potential directions of the German society. Here, SSP1 and SSP5 are used to explore extremes, while SSP2 is used as the main projection. All three narratives deal with challenges towards climate mitigation at three different levels. In contrast, SSP3 and SSP4 lie on the same level on climate mitigation as SSP5 and SSP1 respectively, as outlined in O'Neill et al. [65]. A visualisation of the scenarios can be seen in Fig. 4.

#### 3.5.2 Scenario characteristics: electrification

The degree of electrification of the transport sector implemented in the scenario of low electrification, LoEl, is based on the projections of electrification within the EL95 scenario by Dena et al. [22]. In the construction of the scenario, all plugin hybrid EVs in the EL95 study were assumed to consume solely fuels for combustion. The scenario of medium electrification, MidEl, is based on the shares of electrification of the transport sector assumed in KN2050 by Prognos et al. [25]. Lastly, the scenario of rapid electrification, HiEl, acts as an optimistic scenario regarding electrification, illustrating the absolutely minimum demand for RFs. The shares of electrification within the industrial process heat are based on [66] and [67]. The maximum shares of electrification are presented in Table 4.

**Table 4:** Maximum degrees of electrification as modelled in different scenarios.

Process	LoEl	MidEl	HiEl
Cars	64%	95%	100%
Vans	64%	90%	100%
Trucks	25%	70%	100%
Busses	64%	90%	100%
Trains	100%	100%	100%
Ships	0%	0%	0%
Domestic airplanes	0%	50%	100%
International airplanes	0%	0%	0%
Process heat in aluminum industry	60%	90%	95%
Process heat in other subsectors of industry	30%	80%	95%

As progress in electrification of technologies currently happens at rapid rate for many transport modes, including cars [68], heavy road transport [24], [69] and even airplanes [70], [71], hence the creation of the HiEl2 scenario was a necessary investigation. However, it must be underlined that this scenario assumes an optimistic development in electrification, which is rarely observed among German studies.

#### 3.5.3 Summary of scenarios

To summarise the scenarios, Fig. 4 visualise the relation between electrification levels and socioeconomic developments, which are defining the five scenarios. Furthermore, the main characteristics for each of the scenarios are detailed in Table 5.

**Table 5:** Scenarios and pathways analysed in this paper.

Scenario name	Characteristics
LoEl2	Low rate of electrification assumed in transport and industry sector as based on EL95 by Dena et al. [22]. Demand projections are based on SSP2.
MidEl2	Medium rate of electrification assumed in transport and industry sector as based on KN2050 by Prognos et al. [25]. Demand projections are based on SSP2.
HiEl2	Nearly complete electrification assumed in transport and industry sector with the exception of international aviation and shipping. Demand projections are based on SSP2.
MidEl1	Medium rate of electrification assumed in transport and industry sector as based on KN2050 by Prognos et al. [25]. Demand projections are based on SSP1.
MidEl5	Medium rate of electrification assumed in transport and industry sector as based on KN2050 by Prognos et al. [25]. Demand projections are based on SSP5.

## 4 Results

### 4.1 Total final energy consumption and system costs for the different scenarios

**Figure 5:**
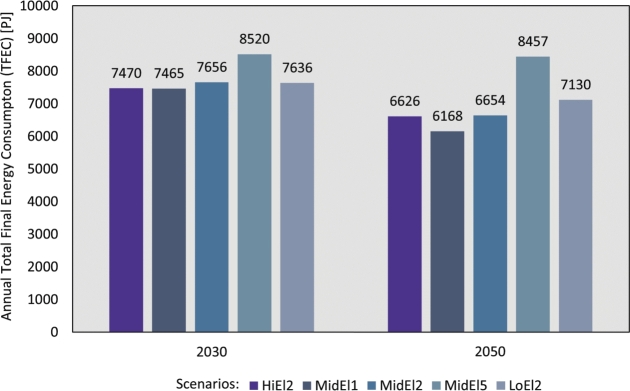
Total final energy consumption across scenarios and pathways analyses in 2030 and 2050.

The Total Final Energy Consumption (TFEC) in Germany for 2030 and 2050 varies depending on the considered electrification scenarios and SSP pathways, as illustrated in Fig. 5.

In general, the results accentuate that increasing electrification level generally yields decreasing TFEC, and that the TFEC varies according to the socioeconomic factors determined by the SSPs. The scenario with the lowest TFEC in 2050 is, therefore, MidEl1, with a TFEC at 6168 PJ, while MidEl5 has the highest demand at 8457 PJ. The main scenario, MidEl2, is in-between these two pathways, with a TFEC at 6654 PJ in 2050. Thus, the socioeconomic mitigation variation results in a variation of the TFEC that is either 7% lower or 27% higher compared to the main MidEl2 scenario.

Despite the increase in energy demand drivers, such as GDP and population, the TFEC decreases over time in all scenarios with the exception of MidEl5. This reduction can be attributed to several factors, such as, energy-saving measures, implementation of energy efficiency, and a shift towards more efficient electric technologies, all of which plays a crucial role in reducing TFEC.

**Figure 6:**
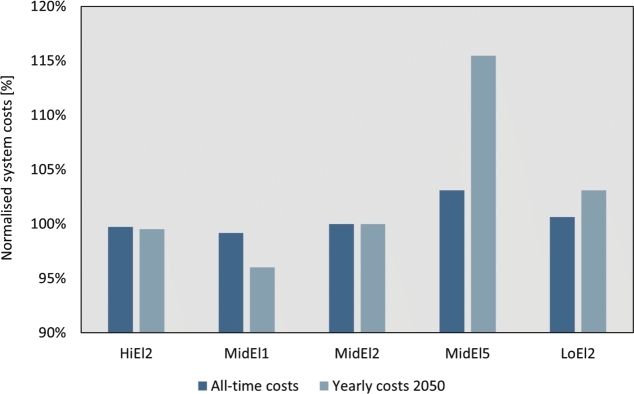
Costs of the energy system across scenarios and pathway analyses.

In addition to the TFEC, we compare the normalised system costs to supply the energy services of the different scenarios. In Fig. 6, it can be seen that the economic gain of a high level of electrification is barely visible, whereas the profit of working towards lower energy demands as seen in MidEl1 is quite significant. In Fig. 6, we show the average all-time system cost and compare it to the 2050 annual system cost to highlight the dynamics related to system costs. The results show that the MidEl5 scenario with high consumption is 15% more expensive on a yearly basis and 4% more expensive on the entire period of modelling compared to the baseline (MidEl2 scenario). These results can be explained by higher energy consumption, and a need for investing and utilising more generation plants compared to other scenarios.

### 4.2 Sector-wise renewable fuel consumption across scenarios

**Figure 7:**
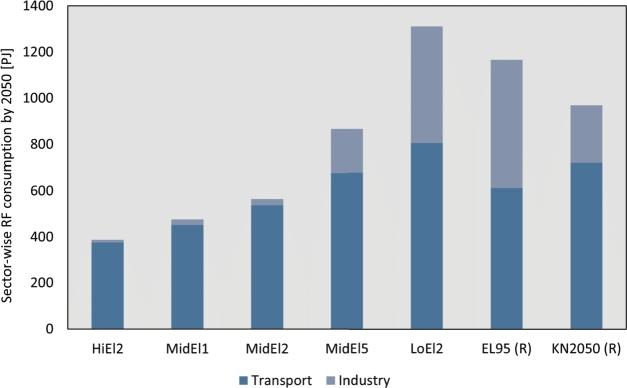
Sector-wise consumption in all scenarios and pathway analyses. For comparison, the results of study [22] and [25] are labelled with (R).

The consumption of RF across different end-use sectors in 2050 is provided in Fig. 7. In all scenarios, the transport sector is the predominant consumer of RFs, constituting between 61.5% and 97.5% of the RF demand. In the LoEl2 scenario, the RF demand is 132% higher than the consumption in the MidEl2 base scenario (564 PJ RF), while in the high electrification scenario (HiEl2), the RF demand is 31% lower than that of MidEl2.

The impact of the SSP pathways is also evident. Indeed, MidEl5 exhibits a 54% higher demand for RFs compared to MidEl2, while MidEl1 has a consumption approximately 16% lower than MidEl2. Furthermore, the increased RF consumption is associated with a higher share of RFs used in the industry sector.

To compare our results with the literature, reference data from the studies conducted by Dena [22] and Prognos [25] are presented in Fig. 7. The comparisons show that the results obtained in this study are in line with existing literature. In detail, the restrictions of the electrification in LoEl2 are based on [67] fit well with the study of comparison. The RF consumption in the industry sector of LoEl2 aligns with the results of the EL95 study [22]. However, the RF demand in MidEl2 is lower compared to the KN2050 study [25]. Specifically, the RF demand in the transport sector found in the MidEl2 is slightly lower than the estimates from KN2050 [25] and EL95 [22]. On the other hand, the demand in the transport sector for LoEl2 is higher than that of EL95, which may be attributed to the specific assumptions made in the scenario descriptions. It is worth noting that HiEl2, with the most liberal electrification constraints, exhibit the lowest RF demand compared to the other scenarios analyzed in this study.

**Figure 8:**
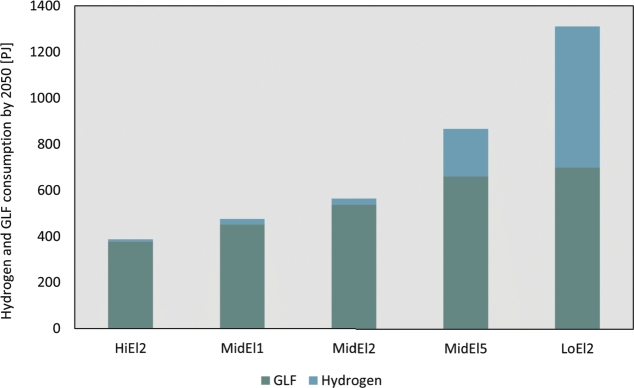
Division of consumption of RFs in 2050 across scenarios and pathway analyses.

Fig. 8 provides a visual representation of the division of RFs consumed in 2050 across different scenarios. In general, GLF are predominantly consumed in heavy transport, including shipping and aviation, while hydrogen is primarily consumed in the industrial sector.

The use of hydrogen as a means to decarbonise the industry is identified as a viable solution. However, the levels of hydrogen consumption vary significantly depending on the degree of electrification. The range spans from 10 PJ in the HiEl2 scenario to 611 PJ in the LoEl2 scenario. Additionally, there is a difference of 183 PJ in hydrogen consumption between SSP1 and SSP5, reflecting the influence of different socio-economic pathways. These variations in hydrogen consumption levels highlight the uncertainties surrounding future scenarios. They also emphasize the need to carefully consider and formulate hydrogen strategies, including the role of self-sufficiency versus importing hydrogen, as discussed in [7].

The utilisation of biofuels is constrained due to competition for scarce biomass resources, primarily in the industrial sector. According to TIMES-DE, it is more cost-effective to allocate a significant portion of biomass as process-heat in the industry with carbon capture, leveraging the resulting negative CO_2_ emissions. As illustrated in Fig. 10, this strategy allows for the utilization of a certain amount of fossil fuels, thus justifying the aggregation of biofuels and E-fuels into the category of GLFs.

**Figure 9:**
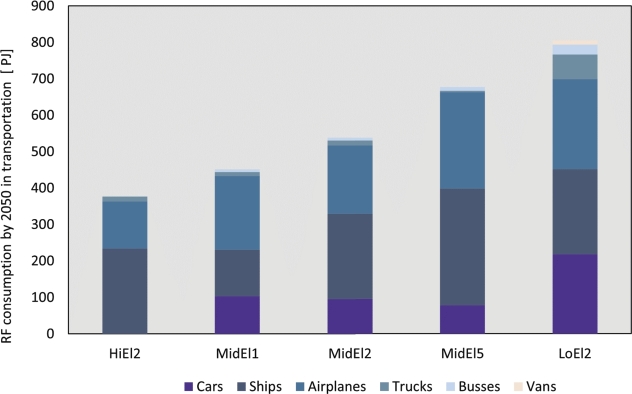
Final consumption of RFs by vehicle in 2050 across scenarios and pathway analyses.

Our findings indicate that the decarbonisation of the transport sector is likely to result in a considerable use of RFs. However, it is worth exploring the breakdown of RF consumption within the transport sector to better understand the demand from each type of vehicle. This breakdown is illustrated in Fig. 9.

In the MidEL2 baseline scenario, the main consumers of RFs within the transport sector are cars, ships, and airplanes, accounting for 91% of the total RF consumption. LoEl2 can be seen as a magnification of these consuming vehicle types, while HiEl2 shows no RF consumption in cars and significantly reduced amounts in airplanes and trucks. This result highlights the widely accepted belief that the extent of electrification plays a crucial role in determining the magnitude of RF demand in the future.

Considering the import shares of RFs in Table 6, it stands clear that Germany will require large amounts of imported RFs at fractions ranging from 45%-68% in the highest RF-demanding scenarios. This is due to the import prices used in the model are more beneficial than utilizing domestic production. This will be further debated in the discussion.

### 4.3 Transition pathway and energy flows for the main MidEl2 scenario

**Figure 10:**
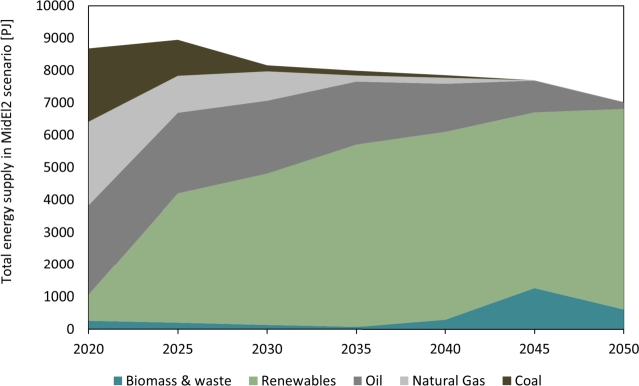
Total energy supply by source for the middle-of-the-road scenario MidEl2.

Fig. 10 presents an overview of the energy transition pathway for in the main MidEl2 scenario, showcasing the shift from a fossil fuel-based to a renewable-based energy system. The transition towards renewables is evident, with a rapid deployment observed in 2025, resulting in the substitution of coal and natural gas, primarily in the electricity and heat sectors. The share of renewables continues to increase towards 2050, leading to a fully renewable-based commercial and residential sector. By 2050, only minimal amounts of oil products and natural gas are utilized in the transport and electricity generation sectors.

Furthermore, the total energy supply experiences a decline over the years due to the move-away from traditional generation methods, which entail significant conversion losses, towards RE sources. This transition is also supported by improved energy efficiency achieved through the adoption of technologies such as heat pumps and electric mobility. Fig. 5 demonstrates a similar trend, except for the MidEl5 scenario.

The motivation behind this energy transition towards higher shares of renewables stems from carbon emission reduction targets and the declining costs of green energy technologies. These factors contribute to the growing competitiveness of renewable energy sources when compared to traditional alternatives.

## 5 Discussion

### 5.1 Key insights and policy implications

This paper investigates the demand for RFs in three different electrification scenarios and conducts additional pathway analyses considering socioeconomic dynamics. Consistent with previous literature [22], the findings confirm that the degree of electrification plays a crucial role in shaping RF demand. Notably, this study reveals a significant effect, resulting in a 60% shift in RF demand compared to the base scenario, depending on the chosen electrification pathway. Furthermore, as electrification increases, Germany experiences a decrease in TFEC. These findings emphasize the potential advantages of electrification in reducing TFEC, reinforcing the significance of incorporating electrification as a strategy to attain sustainable energy objectives. Policymakers should therefore prioritize policies that incentivise the electrification of various sectors, such as transportation and industry, to accelerate the energy transition.

Simultaneously, a noteworthy effect on RF demands and TFEC emerges from the results of the pathway analyses focusing on socioeconomic dynamics, such as GDP and population growth and behavioural change, which aligns with the findings of Chapman [72] and Oxley et al. [73]. Surprisingly, this crucial aspect of demand-side considerations often remains overlooked in mainstream energy scenarios and policy discussions. Nonetheless, there is a growing recognition that reducing demand could be a desirable goal and an impending reality, particularly in light of the current spike in electricity prices [74], leading to decreased energy consumption [28]. Even though the prices most likely will even out over time, such shock events may set their traces in both the demand side [75] and the supply side. The supply side responds by investing in energy sources that will reduce the dependency upon especially Russia [76] pushed by the EU [77], which will aid in lowering the electricity prices.

In this context, policymakers should consider the influence of socioeconomic factors and prioritize energy efficiency programs and regulations to encourage energy conservation in residential, commercial, and industrial sectors. This might include energy efficiency standards for appliances, promoting energy audits, and providing incentives for energy-efficient renovations. Such a focus can contribute to reducing RF demand and further reduce TFEC.

Furthermore, our analysis highlights that the transport sector emerges as the primary consumer of RFs, accounting for a significant share ranging from 61.5% to 97.5% across scenarios. On the other hand, the consumption of RFs in the industry sector shows an upward trend with increased energy demand and decreased electrification levels. It is notable that heavy transport predominantly relies on GLFs, while hydrogen finds its primary use in the industrial sector or in passenger cars.

Taken together, the study highlights the importance of constructing and investigating electrification scenarios, although the assumption of complete electrification of road transport and domestic aviation by 2050, as in HiEl2, may prove to be quite optimistic. Nevertheless, the current estimation of RF demand brought forward in the Climate Action Scenarios by the German Federal Ministry for the Environment, Nature Conservation and Nuclear Safety of approximately 400 PJ [3] equals the demand of the highest electrification scenario explored in this paper. This scenario, as previously discussed, is an optimistic scenario regarding electrification, why policymakers may need to adjust the expected demands in order for companies to design their business plans. Considering the higher costs of RFs compared to fossil fuels [14], the German Federal Ministry for the Environment, Nature Conservation and Nuclear Safety must either work towards a rapid electrification, anticipate a lower demand in the future or assume a drastic drop in prices of carbon sinks to incorporate the climate neutrality goal by 2045. It will most likely be a combination of the above-mentioned. Although not explicitly investigated in this paper, it is assumed that when applying a different SSP to one of the electrification scenarios that are presented without SSP variations (HiEl2 and LoEl2), an equal factorial shift of size and distribution of demand is expected. This could potentially lead to a RF demand of 380 PJ in the case of high electrification and low demand projections.

When comparing to the literature, the base scenario, MidEl2, is in line with the KN2050 scenario [25], from which the rate of electrification within MidEl2 is inspired. However, when comparing the inspiration for LoEl2, EL95 [22], with LoEl2 itself, the results exhibit some difference. This could either be caused by different drivers of demand or by the fact the all plugin hybrid EVs (PHEVs) of EL95 are assumed to be consuming solely combustion fuel. In general, when comparing our results across scenarios to the work of Hansen et al. [36] of 2019, the results line up quite alike. In their three most likely scenarios, they project an RF demand ranging between 515 PJ and 970 PJ, where hydrogen accounts for 0 PJ to 515 PJ. This demand is exclusively attributed to the transport sector since they assume the industry relies entirely on solid biomass for heating processes. A broader analysis was conducted by Blanco et al. in 2018 [11], covering the entire EU and employing a diverse set of scenarios focused on various RFs. In their study, the primary demand for RFs is identified within the industrial sector and heavy transport. It's also evident that achieving higher levels of CO_2_ neutrality leads to an increased demand for RFs.

Existing reviews like Scheller et al. [7] underscores our results in terms of the import share for RF of far more than 50% for Germany in 2050. Future prospects indicate a dynamic market for hydrogen or synthetic fuels, detailing both potential importers and exporters [78]. Within Europe, studies identify hydrogen corridors from the solar-rich southern regions and wind-rich coastal regions towards the centre of Europe [79], [80]. Beyond the European borders, regions such as Australia, the Middle East, and North Africa stand out as prospective exporters, largely due to their abundant sunshine hours. On the other hand, Germany and Japan are noted as importers [81]. Wiese et al. [82] also analyse the imports of hydrogen, methane, and synthetic fuel energy carriers in the context of future German energy systems and conclude that there is a high variance in synthetic fuel import.

### 5.2 Limitations and further work

Given the geopolitical situation resulting in increasing energy prices, including new natural gas prices could yield interesting results. This paper utilises a projection from 2021, but the reopening of society after Covid-19 and the invasion of Ukraine has led to serious modification of the energy market [83]; [84]. Such higher prices could either lead to faster electrification or to an even lower demand of energy [75], why the introduction of elasticity of demand within the model could be an interesting parameter, as well.

In inspiration of the work done by Gaur et al. [31] on their scenario of low energy demand (LED), further work of this study could include more detailed energy savings, expanding from the parameters of GDP, population and sector-wise consumption. As an example, the investigation of the effect of transition towards public transport and the lowering of temperature within houses by 2 °C could be next step.

Another interesting inclusion could be that of a larger region, modelling the entire northern part of Europe as to investigate the competition between countries producing and consuming RFs. In this way, a flow of trades of RFs could be visualised, which could help locating the countries of greatest potential for production and demand and prepare and encourage politicians of these countries to invest in the required infrastructure.

## 6 Conclusion

While achieving climate mitigation targets often involves increasing electrification in sectors that currently emit substantial amounts of CO_2_ equivalents, this study's findings offer valuable insights into future energy consumption and renewable fuel demand in Germany. These insights emphasize the significance of electrification as well as the influence of socioeconomic dynamics. It is worth noting that efficiency measures resulting from socioeconomic pathways have a slightly smaller impact compared to technological progress on the demand for renewable fuel and the total final energy consumption. However, their impact is still significant. These findings challenge the notion that climate mitigation should rely solely on technological advancements and underscore the importance of adopting a holistic approach that encompasses behavioural changes. Such an approach recognizes the interplay between technology, socioeconomic factors, and individual actions in achieving sustainable outcomes. Our results also indicate that hydrogen in its pure form is primarily consumed in industry and heavy-road transport, while more complex Green Liquid Fuels are consumed in aviation and shipping. Furthermore, the usage of biofuels is found to be rather limited due to competition for biomass resources.

The parameterising of a TIMES-DE model focusing on 2030 and 2050 allowed for an in-depth analysis of the demand for renewable fuels in Germany through the investigated impacts of socioeconomic dynamics as implemented in the socio-economic pathways. These pathways encompass changes in gross domestic product, population, energy efficiency, and behaviour. Results showed that these pathways could increase renewable fuel demand by 54% or decrease it by 16% compared to the baseline scenario of 564 PJ. Electrification scenarios were also examined, indicating a potential increase of 132% or decrease of 31% in renewable fuel demand for slow or rapid electrification, respectively.

Based on our findings, policies aimed at promoting electrification have the potential to significantly decrease energy consumption and mitigate climate challenges. Policymakers should also recognize the impact of socioeconomic factors and prioritize the implementation of energy efficiency programs and regulations across residential, commercial, and industrial sectors.

## CRediT authorship contribution statement

**Jonathan Vincents Eriksen:** Writing – review & editing, Writing – original draft, Visualization, Resources, Methodology, Investigation, Formal analysis, Data curation, Conceptualization. **Sebastian Marco Franz:** Writing – original draft, Methodology, Investigation, Data curation. **Julius Steensberg:** Writing – original draft, Methodology, Investigation, Data curation, Conceptualization. **Adam Vejstrup:** Writing – original draft, Methodology, Investigation, Data curation, Conceptualization. **Mikkel Bosack:** Software, Resources, Methodology, Data curation, Conceptualization. **Rasmus Bramstoft:** Writing – review & editing, Writing – original draft, Visualization, Supervision, Project administration, Investigation, Funding acquisition, Data curation, Conceptualization. **Fabian Scheller:** Writing – review & editing, Writing – original draft, Supervision, Resources, Project administration, Funding acquisition, Formal analysis, Conceptualization.

## Declaration of Competing Interest

The authors declare that they have no known competing financial interests or personal relationships that could have appeared to influence the work reported in this paper.

## Figures and Tables

**Table 6:** Collected results.

Scenario	Total Final Energy Consumption [PJ]	
Year	2030	2050
HiEl2	7470	6626
MidEl1	7465	6168
MidEl2	7656	6654
MidEl5	8520	8457
LoEl2	7636	7130



Scenario	Total Final RFs Consumption [PJ]		Share of RFs to TFEC [%]		Share of RFs Imported [%]	
Year	2030	2050	2030	2050	2030	2050
HiEl2	74	388	1	6	0	52
MidEl1	70	476	1	8	0	55
MidEl2	67	564	1	8	0	45
MidEl5	115	867	1	10	0	68
LoEl2	77	1310	1	18	0	61



Scenario	Hydrogen Consumed [PJ]		GLFs Consumed [PJ]	
Year	2030	2050	2030	2050
HiEl2	0	10	74	378
MidEl1	0	23	70	453
MidEl2	0	26	67	538
MidEl5	0	206	115	661
LoEl2	0	611	77	700



Consumption across sectors [PJ]	IND		TRA	
Year	2030	2050	2030	2050
HiEl2	0	11	74	377
MidEl1	0	23	70	453
MidEl2	0	26	67	538
MidEl5	0	189	115	678
LoEl2	0	505	77	805

## Data Availability

Data used in the preparation of the article can be made available on request by contacting Jonathan Vincents Eriksen at jonathanvincents@gmail.com.

## Appendix A Table of summary

The following Table 6 gives a comprehensive overview of the main findings presented within the result section 4.
